# Genetics in parkinson’s disease: From better disease understanding to machine learning based precision medicine

**DOI:** 10.3389/fmmed.2022.933383

**Published:** 2022-10-03

**Authors:** Mohamed Aborageh, Peter Krawitz, Holger Fröhlich

**Affiliations:** ^1^ Bonn-Aachen International Center for Information Technology (B-IT), Rheinische Friedrich-Wilhelms-Universität Bonn, Bonn, Germany; ^2^ Institute for Genomic Statistics and Bioinformatics, University Hospital Bonn, Bonn, Germany; ^3^ Department of Bioinformatics, Fraunhofer Institute for Algorithms and Scientific Computing (SCAI), Sankt Augustin, Germany

**Keywords:** Parkinson disease, risk, genome-wide association study, machine learning, polygenic risk score

## Abstract

Parkinson’s Disease (PD) is a neurodegenerative disorder with highly heterogeneous phenotypes. Accordingly, it has been challenging to robustly identify genetic factors associated with disease risk, prognosis and therapy response via genome-wide association studies (GWAS). In this review we first provide an overview of existing statistical methods to detect associations between genetic variants and the disease phenotypes in existing PD GWAS. Secondly, we discuss the potential of machine learning approaches to better quantify disease phenotypes and to move beyond disease understanding towards a better-personalized treatment of the disease.

## 1 Introduction

Parkinson’s Disease (PD) is a neurodegenerative disorder (NDD) affecting 7–10 Million patients worldwide. PD patients suffer from motor symptoms like bradykinesia, rigidity, tremor, and postural instability. Speech impairments, characterized by hypokinetic dysarthria, are among the first symptoms (including disruptions in prosody, articulation and, phonation). In addition, non-motor symptoms include cognitive impairment, sleep disorders as well as autonomic and mood dysfunction. The cause of idiopathic PD is unknown, and all currently available treatments (e.g. l-DOPA) are symptomatic. PD has a high subject-to-subject variability of symptoms reflecting disease progression (Poewe et al., 2017).

In recent years, genome-wide association studies (GWAS) have shed light on the polygenic nature of Parkinson’s Disease (PD) (Simón-Sánchez et al., 2009; Satake et al., 2009; Kara et al., 2014; Siitonen et al., 2017; Bandres-Ciga et al., 2019; Nalls et al., 2019). First GWAS aimed to identify mutations in coding regions that could be linked to each neurodegenerative trait. Accordingly, variants associated with *α*-synuclein were detected (Mata et al., 2010), one of the hallmark proteins of the disease. However, a meta-analysis of several studies found more variants with smaller effects to be more common in patients than fully penetrant variants (Tran et al., 2020). In addition, larger cohorts now open the possibility to identify less frequent variants and study the interaction with environmental factors. An example is the 23andMe PD cohort, which was able to identify 17 new risk loci for idiopathic PD (Chang et al., 2017; Nalls et al., 2014). Another example is United Kingdom Biobank (UKB), where other authors were able to demonstrate novel gene-environment interactions (Jacobs et al., 2020).

Despite these successes, unraveling the genetic basis of PD, specifically in its sporadic form, remains challenging:

• PD demonstrates a highly heterogeneous phenotype with different long-term outcomes (Aasly, 2020). Accordingly, it is difficult to find genetic associations. So far most research has focused on risk factors for PD diagnosis, but less attention has been paid to identifying genetic variants associated with different long-term outcomes. Notably, a few papers report on genetic risk factors for cognitive impairment in idiopathic PD (Collins and Williams-Gray, 2016; Amer et al., 2018; Planas-Ballvé and Vilas, 2021).
• Sizes of existing cohorts still impose a statistical challenge to identify rare variants.
• Many genetic variants jointly contribute to the phenotype, possibly in a non-linear manner via gene-gene interactions. Finding the true causal subset of variants is still difficult due to the high dimensionality of the GWAS data, the existence of linkage disequilibrium, and statistically low contributions of rare genetic variants on the population level.
• While in a recent meta-study more than 70 single-nucleotide polymorphisms (SNPs) have been associated with the risk to develop PD, most of them are located in non-coding regions and thus difficult to interpret (Ho et al., 2022).

**FIGURE 1:**
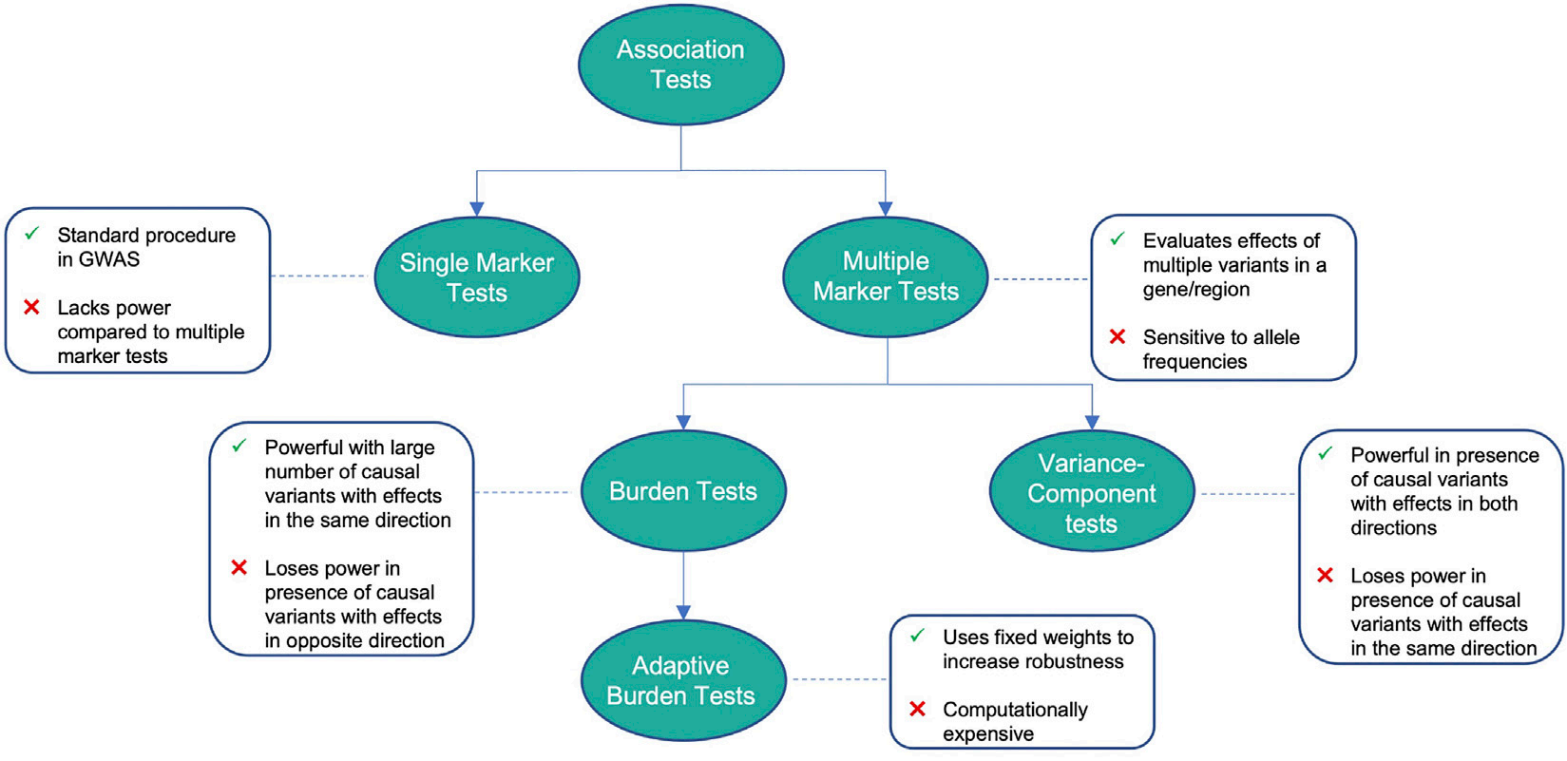
Advantages and disadvantages of different association tests.

In this context the goal of this review is two-fold: First, we provide an overview of existing statistical methods that have been employed to detect associations between genetic variants and the disease phenotype as shown in Figure 1 and Table 1. The second goal of this review is to discuss the potential of machine learning approaches, which could allow to better quantify complex phenotypes and to move beyond disease understanding towards a better personalized treatment of PD in the future. While previous reviews focused on the genetic architecture of PD and discuss associated risk factors (Billingsley et al., 2018), gene-specific polymorphisms (Jiménez-Jiménez et al., 2016), gene-gene and gene-environment interactions (Singh et al., 2014; Dunn et al., 2019), our review has thus a distinguishable methodological focus.

**TABLE 1:** Advantages and disadvantages of methods for variant association testing.

Method	Advantages	Disadvantages
Single-marker tests	Standard method to test for association between variants and traits in GWAS, useful for large sample sizes and common variants with large effect sizes	Less powerful for rare variants with similar effect sizes to common variants, leading to the need for stringent significance levels in scenarios with more rare variants, further reducing its power
Multiple-marker tests	Evaluates the effects of multiple variants in a gene or region, instead of testing for each individually. Has higher power than single-marker tests when variants in a group are associated to the same trait or disease	Highly sensitive to allele frequencies
Burden tests	Powerful in scenarios when a large number of variants are causal with effects in the same direction	Lose power with small numbers of causal variants or in the presence of variants with effects in opposite directions
Adaptive burden tests	Uses fixed weights or thresholds to increase robustness	Computationally intensive
Variance-component tests	Powerful in scenarios with a small fraction of causal variants or in presence of variants with effects in opposite directions	Less powerful with large numbers of causal variants or if their effects are in the same direction
Linkage disequilibrium score regression	Robust against confounders and can be used efficiently with large sample sizes	Despite being computationally less intensive than other genetic correlation methods, a practical setback is the need of processing summary statistics from multiple GWAS which can be time consuming
Mendelian randomization	Overcomes limitations of traditional randomized control trials (RCTs) including proneness to confounders, reverse causation and selection bias	Multiple limitations include pleiotropy where a single variant can produce multiple effects, LD where two variants are statistically associated and tend to be inherited together, and bias of precise estimates of causal effects

## 2 Variant association tests

In 2011, Sun et al. (2011) considered rare variants as single-nucleotide polymorphisms with minor allele frequencies (MAF) less than 0.01, and have larger effects than common variants. However, when combined, the number of low-frequency variants makes them common. According to the multiple rare variant (MRV) hypothesis, cases of common inherited diseases are due to the combined effects of highly-penetrant variants (Bodmer and Bonilla, 2008). The genetic composition of PD is often described by two non-mutually exclusive hypotheses: the common disease common variant (CDCV) hypothesis which describes the genetic basis of PD as a result of a large number of common variants with relatively small effects but combined confer significant disease risk (Pritchard and Cox, 2002), and the common disease rare variant (CDRV) hypothesis which speculates that risk components for complex diseases will be rare genetic variants of small or large effects where highly functional or deleterious alleles may exist. This may be noticeable in late-onset diseases like PD where selective pressures are not profound (Billingsley et al., 2018).

Typically, GWA studies focus on variants with MAF greater than 1–5%, and while they were able to identify several variants with evidence of association to disease risk, these common variants only explain 5%–10% of the disease heritability. This led to the conclusion that disease risk is comprised of both common and rare variants (Schork et al., 2009). Variants located near *SNCA*, *MAPT* genes and low frequency coding variants in *GBA* are validated by GWAS to be statistically significant signals associated with PD (Spencer et al., 2011; Lill et al., 2012; Nalls et al., 2014; Chang et al., 2017).

### 2.1 Single-marker tests

Single-marker testing involves the application of a univariate test for each variant and assessing their significance while using a scaled *p*-value threshold to account for multiple testing (Asimit and Zeggini, 2010). These tests include *X* ^2^, Fisher’s test, Cochran-Armitage (CA) test for trend and regression analysis, be it logistic regression for testing binary traits or linear regression for quantitative traits. Since each variant is tested independently, corrections for multiple testing should be accounted for to control the family-wise error (FWE) which may result in a loss of power. Instead, controlling the false-discovery rate (FDR) by allowing a small proportion of incorrect null hypotheses may result in a gain of power, especially at a larger number of tests.

If we assume *m* number of variants within an *n* number of samples, a regression model can be fit at each of the *m* variants to test their association with a trait. Assuming that *y*_*i*_ is the phenotype for sample *i* and *x*_*ij*_ is the minor allele count of variant *j* for sample *i*, the relationship of variant *i* can be explained by a linear regression model with the following formula:

$$y_{i}=\alpha_{j}+\beta_{j}x_{ij}+\eta_{j}z_{j_{i}}+\varepsilon_{i}$$

Where *z*_*j*_ is a matrix of covariates that may be present, and *ɛ*_*i*_ is an error term representing independent random variables with a mean of 0. For that model, a value of *β*_*j*_ = 0 represents the null hypothesis of no association at variant *j*. For a logistic regression model, *y*_*i*_ is replaced by $\log\left(\frac{p_{i}}{1-p_{i}}\right)$ where *p*_*i*_ is the probability of the trait’s presence.

The *X* ^2^, Fisher’s test, and CA tests construct a 2 × 3 contingency matrix to compare the genotype frequencies between cases and controls, where rows represent disease status and columns represent the three possible genotypes. For *X* ^2^ and Fisher’s tests, a null hypothesis of equal genotype frequencies for both the cases and controls is considered. Usually, Fisher’s test is preferred since it provides exact results of significance, while *X* ^2^ test approximates the results with an accuracy that depends on the sample size, which is not ideal in the case of small samples.

If we represent the genotypes as ordered categories *AA*, *Aa*, and *aa*, the CA test is considered a modification of the *X* ^2^ test to introduce a suspected ordering of the genotype effects and aims to test a linear effect of the minor allele’s copy counts (Slager and Schaid, 2001), which is defined as follows:

$$CA=\frac{n^{2}{\left(\left(n_{Aa}^{0}n^{1}-n_{Aa}^{1}n^{0}\right)+2\left(n_{aa}^{0}n^{1}-n_{aa}^{1}n^{0}\right)\right)}^{2}}{n^{0}n^{1}\left(n_{Aa}\left(n-n_{Aa}\right)+4n_{aa}\left(n-n_{aa}\right)-4n_{Aa}n_{aa}\right)}$$

As mentioned earlier, multiple testing needs to be corrected to control the family-wise error (FWE). The Bonferroni correction is used to test an *m* number of variants while assuming the significance level for the *m* independent hypothesis tests is *α*, using *α*/*m* to calculate the test-specific significance level (Ranstam, 2016). To control the FDR for independent tests, Benjamini & Hochberg (Benjamini and Hochberg, 1995) developed a sequential Bonferroni procedure, where the *m* *p*-values from the individual tests are first ranked: *p*_(1)_ ≤ *p*_(2)_ ≤ ⋯ ≤ *p*_(*m*)_. At FDR level *q*, assume *k* to be the largest *i* such that $P_{\left(i\right)}\leq \frac{i}{m}q$ , then the null-hypothesis is rejected for *p*-values less than *p*_(*k*)_.

A study by Mata et al. (2017) used linear regression to identify genetic variants that may lead to a cognitive decline in PD patients. Eighteen common variants in thirteen genomic regions exceeded the significance threshold for one cognitive test each. However, rare variant analysis did not yield any significance. Another study by Simón-Sánchez et al. (2009) used the Cochran-Armitage test for trend to test associations with PD in European patients. Four SNPs at the *SNCA* locus and three at the *MAPT* locus exceeded Bonferroni-corrected GWAS significance thresholds. An overview about further PD studies and employed statistical tests is provided in Table 2.

**TABLE 2:** Selected studies on risk variant association utilizing multiple techniques.

Author	Method	Objective	Results
Mata et al. (2017)	Single/Multiple-marker, linear regression/SKAT-O	Identify genetic variants leading to cognitive decline in PD patients	Eighteen common variants in thirteen genomic regions exceeded significance threshold
Simón-Sánchez et al. (2009)	Single-marker, Cochran-Armitage trend test	Studying variant association to PD in European patients	Four SNPs within the *SNCA* locus and three at the *MAPT* locus exceeded Bonferroni corrected GWAS significance threshold
Li et al. (2021)	Multivariate linear regression	Test for variant association to age at onset of PD in the Asian population	Identification of a novel intergenic locus which could delay age at onset of PD by 2.43 years
Tan et al. (2021)	Single-marker, linear regression	Identify genetic variants associated with PD progression	Significant association of *APOE* *ϵ*4 tagging variant rs429358 to composite and cognitive progression in PD
Foo et al. (2017)	Multiple logistic regression	Conduct the first Han Chinese GWAS for PD	Presence of some genetic heterogeneity in PD risk between European and East Asian patients
Hernandez et al. (2012)	Multiple-marker, logistic Regression	Identify genetic variants associated with young onset PD in Finnish Patients	Thirteen SNPs that were previously linked to PD showed high significance in the Finnish cohort. However, the study failed to identify any single predominant monogenic causes of the disease in the group
Loesch et al. (2021)	Multiple-marker, logistic regression	Identify PD risk variants in a Latino cohort and describe overlap in genetic structure compared to European ancestry	Genome wide significance shown by *SNCA* locus demonstrating its importance in PD etiology in Latinos
Park et al. (2021)	Multiple-marker, logistic Regression	Identify genetic loci associated with cognitive impairment in patients with sporadic PD	*RYR2* and *CASC17* loci were associated with cognitive impairment based on clinical assessment scores, but none of their SNPs based significance thresholds after Bonferroni correction
Pankratz et al. (2012)	Multiple-marker, logistic Regression	Identification of risk variants associated with PD susceptibility	GWAS significance was reached for previously reported *SNCA*, *MAPT* and *HLA* regions, as well as a novel susceptibility PD locus *RIT2* on chromosome 8
Hill-Burns et al. (2014)	Multiple-marker, logistic regression	Identification of novel PD locus via stratified GWAS study	Identification of a novel locus in chromosome 1p21 in sporadic PD.
Chang et al. (2017)	Multiple-marker, logistic regression	Identification of novel loci associated with PD risk	Identified 17 novel risk loci in a joint analysis of 26,035 cases and 403,190 controls
Hill-Burns et al. (2016)	Multiple-marker, linear regression/Cox regression	Conducting GWAS for age at onset	Two variants, mapped to *LHFPL2* and *TPM1*, were strongly associated to earlier onset PD.
Liu et al. (2011)	Multivariate logistic regression	Identification of risk variants associated to PD in an Ashkenazi Jewish population	The study identified 6 gene regions as candidates for PD using an Ashkenazi Jewish case-control population as discovery set and two other large dataset for replication
Hamza et al. (2010)	Multiple-marker, logistic regression	Conducting a GWAS to identify risk variants in Caucasian population	The study confirmed association with *SNCA* and *MAPT*, replicated *GAK* association and detected novel association with *HLA*, which was replicated in two other datasets
Ryu et al. (2020)	Multiple logistic regression/Cochran-Armitage trend test	Identify genomic variants associated with motor fluctuations and levodopa-induced dyskinesia (LID)	*FAM129B* SNP rs10760490 was nominally associated with motor fluctuations at 5 years after PD onset, while *GALNT14* SNP rs144125291 was significantly associated to occurrence of LID
Rodrigo and Nyholt, (2021)	Multiple-marker, logistic regression	Reanalyzing an ExomeChip-based NeuroX dataset to identify novel, conditional and joint genetic effects associated with PD	Eleven association signals for PD were identified including five novel signals, three of which are driven by low frequencies and two by rare
Blauwendraat et al. (2019)	Multiple-marker, linear regression	Identification of genetic factors associated with age at onset of PD	Results found two GWAS significant signals at known PD risk loci *SNCA* and a protein-coding variant in *TMEM175*, and Bonferroni corrected signals at other known PD loci including *GBA*, *INPP5F/BAG3*, *FAM47E/SCARB2*, and *MCCC1*
Spencer et al. (2011)	Single/Multiple-marker, logistic regression	Performing a GWAS United Kingdom patients to identify novel risk factors associated to PD	Evidence found for PD independent association in 4q22/*SNCA*, weak but consistent association in previously published associated regions 4p15/*BST1*, 4p16/*GAK* and 1q32/*PARK16* and no significant association for previously reported SNP association in 12q12/*LRRK2*
Saad et al. (2011)	Multiple-marker, logistic regression	Performing a three-stage GWAS to identify common PD risk variants in the European population	Significant association of *SNCA* to PD risk, converging evidence of association with PD on 12q24 and confirming associations on 4p15/*BST1*, previously reported in Japanese data
Blauwendraat et al. (2020)	Multiple-marker, logistic/linear regression	Understand whether genetic variants affect penetrance and age at onset of GBA-associated PD and Lewy body dementia (LBD)	Study shows PD and LBD cases with GBA variants often carry other PD associated risk variants that modify disease risk and age at onset
Spataro et al. (2015)	Combined multivariate and collapsing method (CMC)	Study the contribution of rare variants in the etiology of idiopathic PD	The tests showed significance of dominant genes when analyzing code-altering variants only, while they showed significance of recessive genes when analyzing code-altering, putative code-damaging and putative splice-altering variants
Li et al. (2020)	Weighted sum statistic (WSS)/SKAT-O	Study the association of DnaJ homolog C *DNAJC*s in a large Chinese early-onset PD cohort	Several risk variants showed significance in *DNAJC26*, *DNAJC13*, *DNAJC10* and *DNAJC6*, as well as a novel compound heterozygous mutation in *DNAJC6*
Nalls et al. (2019)	SKAT-O	generate summary statistics of genes passing the inclusion criteria of having at least two coding variants	Out of 113 genes, seven showed high significance including *LRRK2* and *GBA*
Siitonen et al. (2017)	SKAT-O	Identify genetic variants associated to early onset PD in Finnish patients	Novel associations were found in the *CEL* region. However, there is a high chance the finding is a false positive as the *CEL* region has multiple indel mutations
Markopoulou et al. (2021)	SKAT	Understanding the contribution of genetic variants at PD risk genes to individual phenotypic charactertistics of PD	Notable findings show association of *LRRK2* with a prior diagnosis of essential tremors, significant association of *NUCKS1* to Unified PD Risk Scale UPDRS-III motor scores and UPDRS-V (H&Y stage) and association of PD risk SNP rs823118 in the same gene to higher MMSE scores

### 2.2 Multiple-marker tests

Multivariate methods can be used as an alternative to testing variants individually by combining information across the variants and testing the multiple variant sites simultaneously. In that case, a multiple-marker test’s power will be higher than that of single-marker tests for multiple moderate SNP effects. Such approaches include Fisher’s method, Hotelling’s *T* ^2^ test, and multiple logistic or linear regression. These tests may be less powerful as they require multiple degrees of freedom.

Fisher’s test combines the results of all *m* single-marker tests, and the test statistic can be represented by $X^{2}=-2\sum_{i=1}^{m}\log\left(p_{i}\right)$ , assuming *p*_*i*_ are the *p*-values obtained from the *m* single-marker tests. However, the test can be anti-conservative when there are dependencies among the *m* single tests.

Multiple regression can be used to test for the association between the variants and the phenotype in tandem instead of fitting *m* regression models at each of the rare variants separately. A simple regression model with no covariates for a binary trait can be represented as follows:

$$y_{i}=\alpha +X\beta_{i}+\varepsilon_{i}$$

where *X* is an *n* × *m* matrix of the minor allele counts for *n* subjects at *m* variants, and *β* is the *m* vector of regression coefficients. By estimating the associations at each variant collectively, the fit requires *m* degrees of freedom for the test statistics of each null hypothesis with *β*_*j*_ = 0 to have *n* − *m* degrees of freedom rather than *n* − 1 as in single-marker tests.

Hotelling’s two-sample *T* ^2^ is a multivariate generalization of the Student’s t-test (Xiong et al., 2002) which can be used for case-control studies. Assume we have *N*_*A*_ affected and $N_{\bar{A}}$ unaffected samples. To calculate the test statistic, consider *X*_*ij*_ and *Y*_*ij*_ as variables defined for the genotype of marker *j* for individual *i* from the case and control groups. For *N*_*A*_ we find

$$X_{ij}=\left\{\begin{array}{ccc} 1, & \text{ if } & aa\\ 0, & \text{ if } & Aa\\ -1, & \text{ if } & AA \end{array}\right.$$

and *Y*_*ij*_ is defined similarly for $N_{\bar{A}}$ . Assume $X_{i}={\left(X_{i1},\ldots ,X_{im}\right)}^{T}$ , *i* = 1, … , *N*_*A*_ for the cases and $Y_{i}={\left(Y_{i1},\ldots ,Y_{ik}\right)}^{T}$ , $i=1,\ldots ,N_{\bar{A}}$ for controls, and after establishing the *X*_*i*_ and *Y*_*i*_’s pooled-sample covariance matrix S, Hotelling’s two-sample *T* ^2^ test statistic can be expressed as

$$T^{2}=\frac{N_{\text{A}}N_{\bar{A}}}{N_{\text{A}}+N_{\bar{A}}}{\left(\bar{X}-\bar{Y}\right)}^{T}S\left(\bar{X}-\bar{Y}\right)$$

and under the null hypothesis, $\frac{N_{A}+N_{\bar{A}}-m-1}{m\left(N_{A}+N_{\bar{A}}-2\right)}T^{2}$ follows an $F_{m,N_{A}}+N_{\bar{A}}-m-1$ distribution.

A drawback to multiple-marker tests is their sensitivity to allele frequencies. A simulation study on rare variants by Li & Leal (Li and Leal, 2008) shows that Hotelling’s *T* ^2^ test is greatly affected by MAF, and shows a reduction in power in cases of increased numbers of rare causal variants.

Li et al. (2021) used multivariate linear regression to test for variant association to age at onset of PD in the Asian population. Results showed a significant effect of a novel intergenic locus rs9783733 that could delay the age at onset in patients by 2.43 years. Another study by Pankratz et al. (2012) used logistic regression to identify genetic variants associated with *p*D. Genome-wide significance was reached for variants in *SNCA*, *MAPT*, *GAK/DGKQ*, *HLA* region and *RIT2*. Additional tests can be found in Table 2.

### 2.3 Burden tests

Aggregation tests can be used to evaluate the combined effects of multiple variants in a gene or region, rather than testing each of them individually. One class of such tests is called burden tests, which collapse information of multiple variants into a single genetic score and test for its association to a trait (Morgenthaler and Thilly, 2007; Li and Leal, 2008; Zawistowski et al., 2010; Morris and Zeggini, 2010; Asimit et al., 2012). By counting minor alleles across all variants in a set, we can summarize the genotype information, and the statistic is represented by:

$$C_{i}=\overset{m}{\underset{j=1}{\sum }}w_{j}G_{ij}$$

where *G*_*ij*_ represents the allele counts of subject *i* at variant *j*, and *w*_*j*_ is the weight for variant *j*.

The summary genetic score *C*_*i*_ can adapt to different assumptions about disease mechanisms. The MZ test (Morris and Zeggini, 2010) utilizes a dominant genetic model instead of an additive one to calculate *C*_*i*_, which is the number of rare variants for which individual *i* carries at least a single copy of the minor allele. As for the cohort allelic sums test (CAST) (Morgenthaler and Thilly, 2007), it assumes an increase in disease risk with the presence of any rare variant, and sets the genetic score *C*_*i*_ = 0 if there are no minor alleles in the region and *C*_*i*_ = 1 otherwise.

We can focus on rare variants by assuming *w*_*j*_ = 1 when the MAF of the variant *j* MAF_*j*_ is smaller than a preset threshold or *w*_*j*_ = 0 if otherwise. We can upweight rare variants by using a continuous weight function. Madsen and Browning (Madsen and Browning, 2009) proposed $w_{j}=1/{\left[MAF_{j}\left(1-MAF_{j}\right)\right]}^{1/2}$ and Wu et al. (2011) proposed the family of Beta densities *w*_*j*_ = *beta* (*MAF*_*j*_, *α*_1_, *α*_2_) which includes the Madsen and Browning weight as a special case. Information on the functional effects of variants can also be used for weight construction.

Outside of the regression framework, several burden approaches have been presented. The combined multivariate and collapsing method (CMC) (Li and Leal, 2008) collapses rare variants as in CAST, but in different MAF categories and calculates the combined effects of the variants using Hoteling’s *t* test. The Madsen and Browning weighted-sum test (WST) (Madsen and Browning, 2009) uses Wilcoxon’s rank-sum test and obtains the *p*-values by permutation.

All rare variants in a set are assumed to be causal and related to a trait with the same direction and magnitude by burden techniques. Breaking such assumptions can result in a significant loss of power (Neale et al., 2011; Lee et al., 2012a).

Spataro et al. (2015) used different collapsing methods, including the CMC and weighted sum tests, to study the contribution of rare variants in the etiology of idiopathic *p*D. The tests showed high significance in a Mendelian group of genes that comprise genes of dominant and recessive inheritance. In dominant genes, the tests showed high significance only when analyzing code-altering variants. As for recessive genes, the tests showed significance for code-altering, putative code-damaging, and putative splice-altering variants. Another study by Li et al. (2020) used the weighted sum statistic (WSS) to study the associations of the DNAJC proteins family by genetic analysis to early onset PD in a large Chinese cohort. The study identified 61 rare variants, two of which showed significance after Bonferroni correction in *DNAJC26*, two in *DNAJC13*, one in *DNAJC10* and one more in *DNAJC6*, as well as a novel compound heterozygous mutation in *DNAJC6*. An overview of further studies using burden tests can be found in Table 2.

### 2.4 Adaptive burden tests

Adaptive methods were developed to address the limitations posed by the traditional burden tests. These methods are robust in presence of null variants and allow for train-increasing or trait-decreasing variants. Han et al. (Han and Pan, 2010) developed a data-adaptive sum test (aSum) that performs a burden test with estimated directions after first estimating the direction of effect for each variant in a marginal model. It assigns *w*_*j*_ = −1 when *β*_*j*_ is likely to be negative and *w*_*j*_ = 1 if not. This approach requires permutation for the *p*-values to be calculated. This procedure is improved in the step-up test (Hoffmann et al., 2010), which uses a model-selection framework that assigns *w*_*j*_ = 0 when a variant is unlikely to be associated, removing it from consideration.

A more direct approach is utilized by the estimated regression coefficient test (EREC) (Lin and Tang, 2011), which uses estimated regression coefficients for each variant as weights. This is based on the assumption that the true regression coefficient *β*_*j*_ is an optimal weight to maximize power. When minor allele counts (MAC) are small, *β*_*j*_ estimates are unstable, and hence the EREC test stabilizes the estimates by adding a small constant to the estimated *β*_*j*_, which might reduce the test’s optimality. The test uses parametric bootstrap to estimate *p*-values because asymptotic approximation of the test statistic is only accurate for very large samples.

The variable threshold (VT) (Price et al., 2010) is an adaptive modification that chooses the best frequency thresholds for rare variant burden testing and calculates *p*-values analytically or by permutation. Using kernel-based adaptive weighting, the kernel-based adaptive cluster (KBAC) (Liu and Leal, 2010) method combines variant classification of non-risk and risk variants with association tests.

As referenced in the previous section, Li et al. (2020) included the aSUM and KBAC tests with the WSS test to study the associations of the DNAJC proteins family to early onset *p*D. Further information and results of the study can be found in Table2.

### 2.5 Variance-component tests

This type of association tests uses a variance-component test within a random-effects model and tests for the association of a group of variants by evaluating the distribution of their genetic effects. These tests include the C-alpha test (Neale et al., 2011), the sequence kernel association test (SKAT) (Wu et al., 2010; Wu et al., 2011), and the sum of squared score test (Pan, 2009). These tests evaluate the distribution of aggregated score test statistics of the individual variants.

SKAT is a non-burden test that uses mixed models and includes the C-alpha test in special cases when covariates are absent, and can also accommodate SNP-SNP interactions. SKAT assumes the regression coefficients *β*_*j*_ are independent and follow a distribution with mean 0 and variance $w_{j}^{2}\tau$ , and tests the hypothesis *H*_0_: *τ* = 0 using a variance-component score test. The SKAT test statistic can be represented as

$$Q_{\text{SKAT}}=\overset{m}{\underset{j=1}{\sum }}w_{j}^{2}S_{j}^{2}$$

which is a weighted sum of squares of the single-variant score statistic *S*_*j*_. Similar to burden tests, SKAT is robust to groups that include variants with both positive and negative effects, as it collapses $S_{j}^{2}$ . When comparing burden and SKAT statistics, it is noted that burden tests collapse the variants first before performing the regression, while SKAT collapses individual variant-test statistics, which explains its robustness to mixed signs of *β* and large fractions of non-causal variants.

While burden tests are not powerful when the target region has several noncausal variants or causal variants of different associations, they can outperform SKAT in cases where a high proportion of causal variants with effects in a similar direction are present. Lee et al, (2012b) proposed a unified test that is optimal in both scenarios and combines both burden tests and SKAT in a single framework. The test statistic of the unified test is

$$Q_{\rho }=\rho Q_{B}+\left(1-\rho \right)Q_{S},0\leq \rho \leq 1$$

which is a weighted average of SKAT and burden tests, which reduces to SKAT when *ρ* = 0 or the burden test when *ρ* = 1.

In their meta genome-wide association study, Nalls et al. (2019) used SKAT-O to generate summary statistics of genes with rare coding variants which had an imputation quality larger than 0.8%. 113 genes passed the inclusion criteria of having at least two coding variants. After Bonferroni correction for the 113 genes, seven significant genes were identified including LRRK2 and GBA. Siitonen et al. (2017) also used SKAT-O in their study to analyze variants associated with early onset PD in Finnish patients. The results showed significant associations to PD in the CEL locus which were not previously identified. However, the validity of the result is questioned by the fact that the CEL region has several indel mutations (Taylor et al., 1991; Siitonen et al., 2017).

### 2.6 Linkage disequilibrium score regression

Linkage Disequilibrium score regression (LDSC) is a method developed by Bulik-Sullivan et al. (2015) that determines if the distribution of a test statistic in GWAS is inflated due to confounding biases or polygenicity. The idea behind LDSC is that variants in linkage disequilibrium (LD) with a causal variant in an association analysis will show elevated test statistics that are proportional to the LD with the causal variant, while elevations due to confounders like cryptic relatedness or population stratification will not correlate with the LD score. LDSC involves using regression techniques to study the relationship between LD scores and test statistics of SNPs obtained from GWAS studies.

Nalls et al. (2019) used LDSC in their GWAS to examine correlations of PD genetics with that of other traits and diseases using data obtained from GWAS available via LD Hub (Zheng et al., 2017) and biomarker GWAS summary statistics on c-reactive protein and cytokine measures. *p*-values obtained from the LDSC were adjusted for FDR to account for multiple testing. The authors found four significant correlations, two of which were positive correlations with intracranial volume and putamen volume, and two negative correlations with tobacco use and educational attainment.

Tirozzi et al. (2020) wanted to investigate the genetic overlap between PD and platelet parameters since associations between both have been established but not thoroughly investigated on a genetic level. The authors applied LDSC to summary statistics of a large independent GWAS conducted on Alzheimer’s disease (AD), PD, and platelet parameters including mean platelet volume (MPV), platelet count (PLT), and platelet distribution width (PDW) (Jansen et al., 2019). The results showed a significant correlation between PDW and PD risk suggesting the existence of genetic overlap and presenting PDW as a new potential biomarker for PD.

Another study by Andersen et al. (2021) investigates how the immune system contributes to pathogenesis in PD, by studying the enrichment of common variant heritability for PD stratified by immune and brain cell types. The authors performed a stratified LDSC (s-LDSC) analysis using full summary statistics from the meta-analysis of PD GWAS by Nalls et al. (2019) and an earlier meta-analysis by Chang et al. (2017). The results found significant enrichment in open chromatin regions of microglia, with further investigation of expression quantitative locus (eQTL) databases showing the *P2RY12* locus to be the most interesting, suggesting it as a microglial gene with PD association signal.

### 2.7 Mendelian randomization

Mendelian Randomization (MR) is a method that uses measured variation in genes of known function to study the causal effects of a modifiable exposure on disease or health-related outcomes (Lawlor et al., 2008). MR studies use genetic variants as instrumental variables (IV) which can be defined as variables that are associated with an outcome only through their robust association with an intermediary variable.

In this context, the aim of MR studies is not to identify genetic variants that are directly associated with the disease but to use the variants as IVs for the modifiable exposure of interest. The genetic variants need to satisfy three assumptions to be considered as IVs in MR studies:

• The variant is associated with the modifiable exposure
• The variant is independent of confounding factors that confound the association of the modifiable exposure to the outcome
• The variant is independent of the outcome given the modifiable exposure and the confounding factors

**FIGURE 2:**
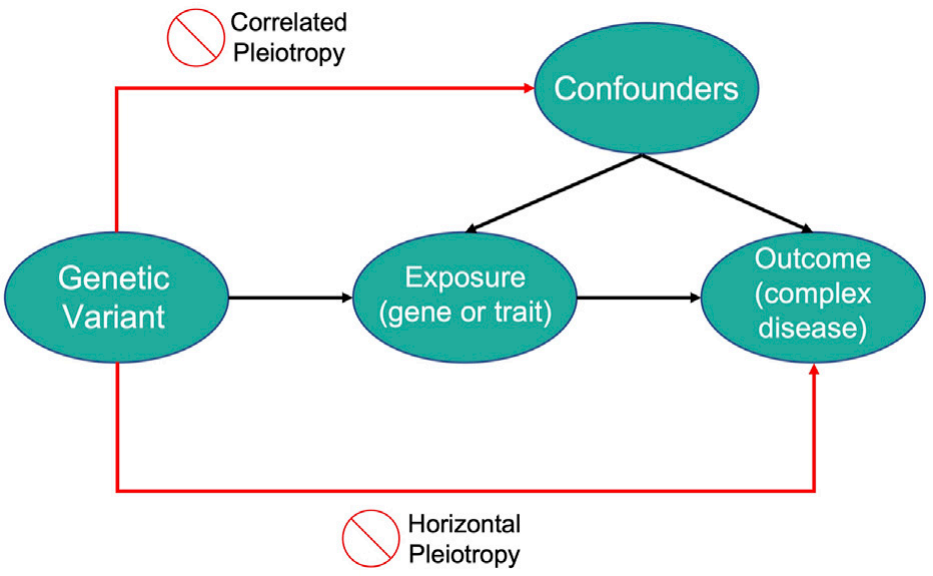
An illustration of Mendelian Randomization framework.

Therefore, genetic variants that explain variations in an exposure can be used as a proxy to explain how changes in that exposure can influence the outcome of a disease of interest. An illustration of the MR framework is shown in Figure 2.

MR was used by Simon et al. (2014) to investigate whether genetic variants that can predict serum urate levels can predict the rate of progression in patients with early PD, on the basis that higher serum urate levels lower the risk of developing *p*D. In this study, the authors used *SLC2A9* gene as an IV, which explains most of the genetically specified variability in serum urate levels but does not have any known direct associations with the central nervous system. The authors then estimated the association between genetically determined urate levels and PD progression using two-stage regression, where they first fitted a generalized linear regression model with urate levels as the dependent variable, and a *SLC2A9* score based on the number of minor alleles at three selected loci, along with potential confounders, as independent variables. Then, a Cox proportional hazards model used the predicted urate levels from the first stage regression as a continuous independent variable to determine its association with PD progression. The results showed that an increase in the number of *SLC2A9* minor alleles is associated with a decreased serum urate level. Also, the rate of PD progression increased with the number of minor *SLC2A9* alleles associated with lower serum urate levels. Genetic variants other than *SLC2A9* did not show any significant association to lower serum urate levels or rapid PD progression. The results suggest that high serum urate levels are protective of rapid progression in early PD.

Similarly, a study by Domenighetti et al. (2022) uses MR to investigate the association between genetically predicted dairy intake and higher PD risk by using the *LCT* lactase gene’s minor allele rs4988235 as an IV, where TT/TC genotypes are associated with lactase persistence and the ability to digest lactose and CC genotype with non-persistence. The authors then used logistic regression to compare the frequency of rs4988235-TC+TT genotypes in patients and controls of European ancestry. Results showed that rs4988235-TC+TT genotypes were more frequent in PD patients than controls, suggesting that higher dairy intake increases PD risk.

Another study by Storm et al. (2021) uses MR to investigate several druggable genes and predict their efficacy as PD drug targets. In this study, the authors considered the expression levels of the druggable genes as the modifiable exposure, while variants associated with expression levels of the genes, called eQTLs, were used as the IVs. The authors sought to use openly available eQTL data for genes under investigation to mimic exposure to corresponding medications (Finan et al., 2017). First, the authors used the cohort collected for the meta-analysis by Nalls et al. (2014). The causal estimates, known as the Wald ratio, were calculated for each SNP, and the ratios were weighted by inverse-variance (IVW) for genes with more than one eQTL available. This identified 31 genes with genetically-determined expression that is highly associated with PD risk. The authors then attempted to replicate the genes with significant association with PD risk in an independent cohort that does not overlap with the original cohort. The authors then used several meta-analysis methods to look for pleiotropy due to confounders including IVW, the MR-Egger intercept test, Cochran’s Q test, and the *I* ^2^ test. Based on the results, the authors propose the genes *CTSB*, *GPNMB*, *CD38*, *RHD*, *IRAK3* and *LMAN1* as drug targets with the strongest MR evidence.

As previously discussed, Nalls et al. (2019) identified correlations of PD genetics with tobacco consumption, educational attainment, and brain volumes using LDSC. The authors used MR to assess the existence of a causal relationship between PD and the traits. The results showed that cognitive performance and educational attainment had a large causal effect on PD risk, while smoking and brain volumes did not have any significant causal relationship.

### 2.8 Multiple testing corrections

Multiple testing is one of the major concerns regarding high-dimensional data which results from simultaneous testing of multiple hypotheses, which if not taken into consideration, may lead to rejecting a true null hypothesis by chance, known as a false discovery. This can be accounted for by controlling an appropriate error rate such as the family-wise error rate (FWE) which is the probability of one or more false discoveries. The classical method of controlling FWE is the Bonferroni method (Bland and Altman, 1995), which is an adjustment made to *p*-values when several tests are performed. To perform a Bonferroni correction, assume the critical *p*-value to be *α*, then divide it by the number of tests made *n*. The new critical *p*-value would then be *α*/*n*, and the statistical power of the study is then calculated based on the newly modified *p*-value.

Another method is the Benjamini–Hochberg method which controls the false discovery rate (FDR) (Benjamini and Hochberg, 1995), known as the expected proportion of false rejections out of all rejections. The Benjamini–Hochberg procedure involves ordering all *p*-values from smallest to largest then assigning a ranking to each one, then calculating the critical *p*-value as (*i*/*m*)*Q*, where *i* is the rank of the *p*-value, *m* is the total number of tests and *Q* is the chosen FDR. The method then checks the largest *p*-value below the critical rate, and considers any smaller values as significant.

## 3 Polygenic risk score

The risk of polygenic disorders such as PD cannot be assessed by information conferred from a single variant, but the total set of risk variants that comprise its genetic architecture is required to provide enough information that can help identify individuals at high-risk (Lewis and Vassos, 2020). An individual’s risk can be assessed using polygenic risk scores (PRS), calculated as the sum of risk alleles an individual carries, each weighted by their relative effect sizes obtained from the GWAS summary statistics (Ibanez et al., 2019), where the result is a score that represents the individual’s genetic load for the disease or trait in question.

In this context linkage disequilibrium (LD) and *p*-values thresholds for individual SNPs have to be considered. Simpler approaches, such as PRSice (Choi and O’Reilly, 2019) and PLINK (Purcell et al., 2007; Gaunt et al., 2007; Chang et al., 2015), only use *p*-value thresholds (clumping + thresholding), whereas more advanced methods, including LDPred (Vilhjálmsson et al., 2015), PRS-CS (Ge et al., 2019), JAMPred (Newcombe et al., 2019), and Lassosum (Mak et al., 2017) additionally take into account based on reference data.

While PRS can provide a simple estimate of the genetic architecture of complex disorders, its additive model generally does not take into account gene-gene interactions (Aschard, 2016). Moreover, the typically required pre-filtering of SNPs implies a focus on more common genetic variants.

The largest meta-analysis was performed by Nalls et al. (2014) and was considered the reference for PD-related PRS before including more data from the 23andME (Chang et al., 2017) meta-analysis. The included PRS were associated with PD status, faster motor and cognitive decline (Paul et al., 2018) and age at onset of disease. Another study by Escott-Price et al. (2015) mentions that only PRS built from SNPs with *p*-values below the significant thresholds were associated with PD, suggesting that the genetic architecture of PD includes several common variants with small effects. Another study by Ibanez et al. (2017) shows that PRS from more significant SNPs are also associated with PD risk. Furthermore, PRS were used to show a higher genetic burden in early-onset PD than in late-onset PD (Escott-Price et al., 2015). More studies with established PRS in the PD field can be found in Table 3. A more detailed review of PRS in the PD field can be found in (Dehestani et al., 2021).

**TABLE 3:** Polygenic Risk Scores listed in the Polygenic Score Catalog (Lambert et al., 2021).

Author	Reported traits	Ancestry Distribution	Number of variants
Pihlstrøm et al. (2016)	Parkinson’s disease, motor decline	European	19
Ibanez et al. (2017)	Parkinson’s disease, age at onset	European	16
Paul et al. (2018)	Parkinson’s disease, cognitive decline, motor decline	European	23
Nalls et al. (2019)	Parkinson’s disease	Multi-ancestry	90, 1805
Bobbili et al. (2020)	Parkinson’s disease	European	43
Liu et al. (2021)	Parkinson’s disease dementia	Multi-ancestry	3
Sia et al. (2021)	Parkinson’s disease	East Asian	6
Chairta et al. (2021)	Parkinson’s disease	European	12

## 4 The perspective of machine learning

### 4.1 Multi-modal data integration

There is an increasing awareness that PD has to be understood as a complex disease, in which aging, (epi-)genetic variants, environmental pollutants/toxins, lifestyle, and comorbidities jointly contribute to the observed phenotype (Espay et al., 2017; Titova and Chaudhuri, 2017). Whereas variants association tests and PRS have helped to gain a better understanding of the genetic basis of PD, developing algorithms for accurate disease risk assessment, diagnosis, prognosis, and treatment response in the context of precision medicine require combining PRS as well as relevant genetic variants with further data modalities. Hence, predictive machine learning models are needed, which can potentially also overcome one of the typical limitations of PRS, namely lacking variant interactions and thus non-linearities. A recent study shows the combined role of PRS, rare high-impact variants, and family history in PD risk (Hassanin et al., 2021). Cope et al. demonstrated that a non-linear machine learning algorithm purely trained on genetic variants can result in dramatically improved prediction performances compared to a classical PRS (Cope et al., 2021). Notably, analysis of the model allowed us to identify an interaction between variants in *TMEM175* (coding for a potassium channel in late endosomes) and *GAPDHP25* (glyceraldehyde-3 phosphate dehydrogenase pseudogene 25), which have been linked to PD (Nalls et al., 2014). Another study by Prashanth et al. (2016) used multimodal features to classify early PD subjects from controls using machine learning models. The authors used non-motor features of Rapid Eye Movement (REM) sleep Behaviour Disorder (RBD) and olfactory loss as well as cerebrospinal fluid (CSF) measurements and dopaminergic imaging markers to classify the patients using Naive Bayes, Support Vector Machine (SVM), Boosted Trees and Random Forest classifiers, where SVM gave the highest performance. Based on the results, the authors suggest that the combination of non-motor, CSF, and imaging features can help in the preclinical diagnosis of PD.

A further example is the use of non-linear unsupervised machine learning algorithms by Emon et al. (2020) to identify patient subgroups by exploring the genetic burden by SNPs in genes that have been previously associated with AD and PD, which allowed for a molecular mechanism based stratification of AD and PD patient sub-types. The authors further investigated clinical outcome measures of the patients to confirm whether the patient clusters were disease-associated or reflected general genetic variations in the population and found the clusters to be associated with different clinical symptoms, pathophysiological brain differences, and biological processes that were enriched only in each of the clusters.

Experiences from neurological conditions other than PD suggest that combinations of PRS, (non-linear) combinations of genetic variants, pathway-level burden scores and a detailed description of the clinical phenotype could allow for a rather accurate prediction of disease risk (Khanna et al., 2018; Birkenbihl et al., 2020) and even clinical drug response (de Jong et al., 2021). Interestingly, in both cases, genetic factors played a comparably small role in the prediction of the clinical outcome. In another study, Makarious et al. (2022) demonstrate the benefits of using multiple data modalities by integrating clinical, genetic, and transcriptomic data in a predictive machine learning framework. Their results showed that integrating multiple data modalities improved PD prediction in mixed populations of cases and controls. They also demonstrated the benefits of using machine learning approaches and the ability to tune the models’ parameters and accommodate nonlinearities, as well as identifying important features that contributed the most to the models’ predictive performance using model explanation methods such as SHAPley Additive exPlanations (SHAP).

### 4.2 Deep phenotyping

A few studies have started to focus on genetic risk factors associated to symptoms of idiopathic PD, including cognitive impairment (Collins and Williams-Gray, 2016; Amer et al., 2018; Planas-Ballvé and Vilas, 2021). In this context, it has to be re-emphasized that PD patients suffer from a whole spectrum of motor and non-motor symptoms. Traditionally, these symptoms are assessed via questionnaires, such as the Unified Parkinson’s Disease Rating Scale (UPDRS), during a patient’s visit to a medical specialist center. The assessment is dependent on the experience of the individual examiner and can thus be subjectively biased. Therefore, during the last years, there has been a strongly growing interest in remote monitoring techniques (RMTs), including wearable sensors and devices (measuring e.g. gait) and smartphone apps (measuring e.g. cognitive abilities). Compared to established questionnaire-based assessments, RMTs offer several potential benefits:

1) They are patient-centric and not biased by a rater’s experience.
2) They allow for monitoring disease symptoms within a patient’s natural at-home environment, potentially 24/7, hence considering the fact that PD symptoms are variable over the daytime. RMT signals can thus be viewed as real-world data.
3) Digital sensing techniques provide an objective measure of a clinical symptom.

Notably, processing of RMT signals requires advanced data analytical techniques, including machine learning (Fröhlich et al., 2022). The outcome is an abstract set of features representing a patient’s phenotype. Following sufficient validation, within clinical studies, these features can result in digital biomarkers, which provide an accurate and quantitative description of PD symptoms. The combination with genetic data thus opens completely new opportunities to identify risk factors for specific PD symptoms, such as cognitive impairment or sleep disturbances. Moreover, machine learning algorithms could potentially be used to combine digital biomarkers with genetic features and other data modalities, including electronic health records, to predict disease risk, prognosis, and response to treatment.

### 4.3 Parkinson’s disease prediction

Multiple studies have used machine learning models to predict PD using different data modalities, analyzing hidden information in data that cannot be interpreted in clinical diagnosis. Wang et al. (2020) investigated the diagnosis of PD based on vowel phonation. Features were obtained from the mPower dataset and improved with additional novel features using a Bayesian correlated *t*-test. The features were then used as input for an SVM model which performed with moderate accuracy. Bhurane et al. (2022) used SVM with a cubic kernel to classify PD patients and healthy controls. Using features extracted from Electroencephalography (ECG) signals, the proposed approach performed with high accuracy.

Chakraborty et al. (2020) used features extracted from 3T T1-MRI scans to detect neurodegeneration in *p*D. Using atlas-based segmentation, eight subcortical structures were segmented from the MRI scans, on which feature extraction was performed to extract textural, morphological, and statistical features. The features were then used to train four different machine learning algorithms: an artificial neural network (ANN), XGBoost model, random forest classifier, and an SVM, where the ANN model performed with the highest accuracy. In another study, Ali et al. (2019) used neural networks to detect PD using features obtained from acoustic analysis of voice signals. Linear discriminant analysis (LDA) was used for dimensionality reduction, and a genetic algorithm (GA) to optimize the hyperparameters of the neural network. Initially, the model performed with accuracy which falls after excluding gender-dependent features to eliminate bias.

Peng et al. (2019) used a three-step method for PD gene prediction. The method, called N2A-SVM, uses the Node2vec algorithm to extract vector representations of each gene in the protein-protein interaction (PPI) network. Then it uses an autoencoder to reduce the dimensions of the obtained vector, and an SVM for classification. The performance of N2A-SVM was tested in comparison to the other methods: random walk with restart (RWR) (Li and Patra, 2010), shortest path length (SPL) (Krauthammer et al., 2004) and Euclidean distance (ED) (Díaz-Uriarte and Alvarez de Andrés, 2006), where N2A-SVM showed the highest performance.

Another study by Rastegar et al. (Ahmadi Rastegar et al., 2019) used machine learning models to assess if serum cytokine levels can be used to predict PD progression. The authors used data from the Michael J Fox Foundation *LRRK2* clinical cohort consortium to assess the variability of inflammatory cytokine levels in patients over a one-year period. Then, the authors used the cytokine measurements with elastic net and random forest models to predict longitudinal clinical outcomes. Using baseline cytokine measurements, random forest models of motor severity showed the best predictive performance, with cytokines *MIP*1*α* and *MCP*1 contributing the most to the predictive model.

## 5 Discussion

The heterogeneous nature of PD imposes specific challenges for finding the underlying genetic causes. We briefly discussed several association tests that were used to identify genetic variants associated with disease risk. Single-marker tests are the simplest approach to studying associations by applying a univariate test to each variant and assessing their significance. However, their statistical power is low for small datasets and requires corrections for multiple testing. These issues were addressed by developing statistical methods that evaluate the associations of multiple variants in specific regions or genes. They are used as a standard method to test for variant association in GWAS, and helped identify several variants associated with PD including *SNCA*, *MAPT*, *GBA* and *HLA* loci as well as others associated with cognitive decline in PD including *APOE* *ϵ*4, *RYR2* and *CASC17* loci.

Burden tests collapse multiple genetic variants into a single genetic score, which is used to test the association to a trait. Since these tests assume all collapsed rare variants to be causal and associated with the trait under study in a similar direction and magnitude of effect, any changes in said assumptions lead to a loss in their statistical power. Adaptive burden tests address these limitations as they require fewer assumptions about the genetic architecture at each locus, and hence they are suitable in the presence of null variants and trait-increasing or decreasing variants. However, adaptive tests are two-step procedures that may require regression coefficient estimation of individual variants as a first step and can be unstable for rare variants. They also estimate *p*-values by computationally intensive permutation. The use of burden tests helped us understand the role of different variant types in the etiology of idiopathic PD, and the identified four mendelian mutations of *LRRK2* and *PARK2* loci in idiopathic PD cases (Spataro et al., 2015). Adaptive tests were also used to study the associations of DNAJC proteins family with early onset PD (Li et al., 2020).

Variance-component tests evaluate the distribution of genetic effects for groups of variants to test for their association. Instead of aggregating the variants, they assess the distribution of each of the variants’ aggregated score test statistics. Variance-component tests are more powerful than burden tests if the genetic region under study has many non-causal variants or variants with different directions of association, while burden tests are more powerful when there are more causal variants with the same direction of association. SKAT-O combines both burden tests and SKAT in a single framework but can be less powerful than any of its components if their underlying assumptions are largely true. Nalls et al. (2019) used SKAT-O in their meta GWAS to identify genes with two or more rare coding variants, and 7 significant genes: *LRRK2*, *GBA*, *CATSPER3*, *LAMB2*, *LOC442028*, *NFKB2* and *SCARB2*. SKAT has also been used to study the association of genetic variants to individual phenotypic characteristics of PD, including motor and cognitive functions (Markopoulou et al., 2021).

LD score regression helped researchers distinguish whether inflated GWAS test statistic distributions are due to variants in LD with a causal variant or due to confounding bias or polygenicity. LDSC has been used to examine correlations of PD genetics with different traits, including brain measurements, blood measurements, habitual behaviors, and immune system activity in different cell types (Nalls et al., 2019; Tirozzi et al., 2020; Andersen et al., 2021).

Mendelian Randomization helped understand the causal effects of modifiable exposures on *p*D. The method uses the genetic variants as instrumental variables in statistical analysis to describe the relationship between the disease and the modifiable exposure of interest. MR was used to investigate the relationship between PD and serum urate levels, suggesting that elevated urate levels are protective of rapid progression in early PD (Simon et al., 2014). MR was used as well to investigate the relationship with lactose tolerance in different PD patient populations, suggesting that high tolerance and increased dairy intake elevate PD risk (Domenighetti et al., 2022). MR also helped propose druggable targets by investigating the expression levels of druggable genes and using them as the modifiable exposure of interest (Storm et al., 2021).

PRS have opened the possibility to assess disease risk on an individual basis rather than purely on the average population level. Limitations of PRS include their additive nature, which neglects gene-gene interactions, and the focus on more common genetic variants. Machine learning models can mitigate this limitation and additionally include further data modalities, such as other molecular and phenotypic data. In this context, electronic health records, as well as digital biomarkers, could help to longitudinally and more objectively characterize disease symptoms. The main challenge with the use of machine learning models is, however, their difficult interpretation, specifically in the case of neural networks. Novel approaches coming from the field of Explainable AI (XAI) could here provide a solution (Linardatos et al., 2020; Arrieta et al., 2020).

In summary, novel methodological developments are necessary to deepen the understanding of the genetic basis of PD and to transfer these insights into better individualized treatment of PD in the future.
